# Performance of a Novel Worm-Assisted Membrane Bioelectrochemical System: Electricity Recovery, Sludge Reduction, and Membrane Fouling Mitigation

**DOI:** 10.3390/membranes16010002

**Published:** 2025-12-22

**Authors:** Chenyu Ding, Xin Guo, Weiye Bian, Zhipeng Li, Yang Li, Hongjie Wang, Hui Li

**Affiliations:** 1 Hebei Key Laboratory of Close-to-Nature Restoration Technology of Wetlands, School of Eco-Environment, Hebei University, Baoding 071002, China; 18655175391@163.com (C.D.); 17772524307@163.com (X.G.); hbubianwy@163.com (W.B.); 16613120732@163.com (Y.L.); 2 School of Marine Science and Technology, Harbin Institute of Technology at Weihai, Weihai 264209, China; lizhipengcn@hit.edu.cn; 3 Engineering Research Center of Ecological Safety and Conservation in Beijing-Tianjin-Hebei (Xiong’an New Area) of MOE, Baoding 071002, China

**Keywords:** membrane bioreactor, wastewater treatment, energy recovery, worm predation, membrane fouling, XDLVO theory

## Abstract

This study developed a novel worm-assisted membrane bioelectrochemical reactor (W-MBER) that integrates aquatic worms and a single-chamber sediment microbial fuel cell into a membrane bioreactor (MBR) to address challenges in energy recovery, sludge reduction, and membrane fouling. The system achieved a stable output of 290 mV at an external resistance of 250 Ω and a maximum power density of 0.013 W/m^2^ while maintaining high removal efficiencies for chemical oxygen demand (93.57%) and ammonia nitrogen (98.61%). Furthermore, the TN removal efficiency was 12.93% higher than that in the conventional MBR (C-MBR), attributed to the anodic anoxic microenvironment. The synergy of worm predation and the bioelectrochemical process reduced sludge production by 28.51% and extended the filtration cycle by 43.75%, indicating significant sludge reduction and membrane fouling mitigation. Mechanistic analysis revealed that the W-MBER system decreased protein content and protein/polysaccharide ratios in soluble microbial products (SMPs) and extracellular polymeric substances (EPSs), and the hydrophobicity of SMPs, EPSs, and sludge flocs was reduced, resulting in a lower free energy for their interaction with membrane. The foulants in the W-MBER encountered higher energy barriers and lower secondary energy minimums when approaching the membrane, indicating a lower membrane fouling propensity. These results demonstrate the promise of W-MBER for sustainable wastewater treatment.

## 1. Introduction

Membrane bioreactors (MBRs), which integrate activated sludge treatment with membrane filtration, provide an effective solution for the treatment of diverse wastewater streams [1]. However, membrane fouling in MBRs significantly increases operational costs and represents one of the primary bottlenecks limiting its widespread application. Additionally, increasingly stringent environmental regulations on sludge discharge have made excess sludge management a critical challenge in aerobic MBR operations.

Worm predation has emerged as a sustainable biological approach to achieve sludge reduction by extending conventional wastewater treatment food chain with the introduction of higher organisms that consume bacterial biomass [2]. The effectiveness of worm predation for sludge reduction hinges on the specific physiological and ecological roles of aquatic oligochaetes. These benthic worms primarily function as deposit feeders, consuming settled sludge flocs, bacteria, and organic detritus. Their grazing activity directly reduces biomass volume through assimilation and, more significantly, through physical disintegration of flocs and biostimulation via selective feeding and bioturbation [3]. This process alters the microbial community structure and accelerates the mineralization of organic solids, effectively extending the food chain and reducing net sludge yield. Previous research in worm reactors have demonstrated sludge reduction efficiency of 34.4%, attributed to these mechanisms [2]. Crucially, worm activity has also been linked to modifications in sludge properties, including a decrease in the concentration and a shift in the composition of SMPs and EPS, which are key membrane foulants [4]. The direct biomass consumption and modification of foulant properties by worms provides a strong rationale for their integration into MBR systems to address both sludge yield and membrane fouling simultaneously. Some studies have employed the worm predation reactor as a bypass for sludge reduction treatment, achieving efficient sludge reduction and membrane fouling mitigation [5]. However, the bypass operation of the worm predation reactor necessitates sludge recirculation, leading to increased operational complexity and limited automation capability. How to integrate worm predation with the MBRs in situ to construct an integrated worm predation MBR system while maintaining effluent quality has emerged as a critical challenge resolution.

The integration of microbial fuel cells (MFCs) into MBRs has recently attracted considerable attention as a sustainable, external energy-free strategy for in situ membrane fouling mitigation [6,7,8]. Research has shown that MFCs can effectively control membrane fouling in MBRs through electrophoretic effects and electrical stimulation-induced sludge modification during electricity generation [7,8]. Typically, electricity generation performance is considered as a key factor in fouling control [9,10]. Enhancing the electricity generation performance of the MFC can effectively improve the membrane fouling control effect and electrical energy recovery efficiency. However, most research has focused on energy recovery from wastewater, while research on sludge treatment and resource recovery from sludge is relatively scarce [11]. Therefore, enhancing energy recovery from sludge is a key area that warrants further investigation. Sediment MFCs can utilize excess sludge as substrate sediment, converting the chemical energy in sludge into electrical energy [12]. Additionally, Studies have shown that inoculating worms into the sediment can effectively enhance power generation performance [13]. Meanwhile, research has also revealed that worm predation can simultaneously achieve sludge reduction and improve sludge settling properties while decreasing the content of key membrane foulants [14]. Consequently, the in situ integration of worm-enhanced sediment MFCs within MBRs holds promise for simultaneously achieving in situ sludge reduction, efficient energy recovery, and effective membrane fouling control in MBRs.

In this study, a novel worm-assisted membrane bioelectrochemical reactor (W-MBER) was constructed by integrating aquatic worms and a microbial fuel cell into a membrane bioreactor for enhanced electricity recovery, sludge reduction, and membrane fouling mitigation. A conventional MBR (C-MBR), operated in parallel without worms or an MFC component, served as the control to unequivocally evaluate the performance enhancement attributable to the integrated “worm-electrochemical” synergy. This study investigated the following four aspects: (1) the electrochemical performance of the W-MBER, (2) co-effects of the worm predation and MFC on the wastewater treatment and membrane fouling in the W-MBER, (3) variation in the sludge properties and microbial community structure in the W-MBER, (4) mechanisms of membrane fouling mitigation in the W-MBER.

## 2. Materials and Methods

### 2.1. Experimental Materials and Equipment

#### 2.1.1. Source of Wastewater and Sludge

The activated sludge was collected from the Yindingzhuang wastewater treatment plant (Baoding, Hebei Province, China) and used as the original inoculum for the reactors. Prior to inoculation into the MBRs, the sludge was sieved to remove the impurities and acclimatized for 15 days in a sequencing batch reactor (SBR) using synthetic domestic wastewater. The reactor was maintained under the following conditions: dissolved oxygen concentration above 2 mg/L, influent pH near neutral, temperature of 25 °C, a reaction time of 20 h per cycle, a sedimentation period of 2 h, and a 2 h phase for emptying and refilling prior to the next operational cycle. The synthetic wastewater was prepared using glucose, K_2_HPO_4_ and NH_4_Cl as the carbon, phosphorus, and nitrogen sources, respectively, resulting the following concentrations: COD, 319.90 ± 8.50 mg/L; NH_4_^+^-N, 39.63 ± 1.36 mg/L; TN, 40.55 ± 0.71 mg/L; and TP, 7.54 ± 0.71 mg/L.

#### 2.1.2. Construction of the W-MBER System

**Figure 1:**
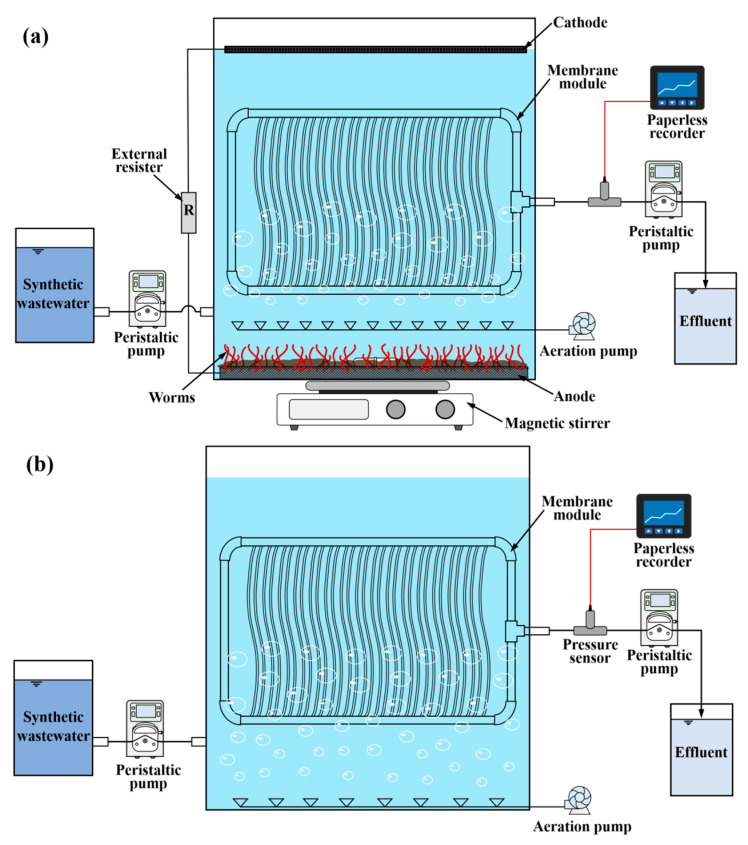
Schematic diagrams of the (**a**) W-MBER and (**b**) C-MBR.

The configuration of the W-MBER system is illustrated in Figure 1a. The W-MBER reactor was constructed from a cylindrical plexiglass tank with dimensions of 17 cm in internal diameter and 30 cm in height, and a working volume was 4.5 L. It was equipped with an anode electrode, a cathode electrode, an aerator, and a membrane module. The anode was made of disc-shaped carbon felt with a thickness of 2 cm and a diameter of 16.5 cm, positioned at the bottom of the reactor. It was selected not only for its high specific surface area and conductivity, which favor electroactive biofilm growth, but also for its three-dimensional porous structure, which provides a suitable protective habitat for the inoculated worms. The cathode, also composed of carbon felt with a thickness of 0.5 cm and a diameter of 10 cm, floated on the liquid surface. The anode and cathode were connected via an external resistor of 250 Ω with copper wire. An aeration device was installed 2 cm above the anode, with the aeration rate controlled at 0.25 m^3^/d to maintain the dissolved oxygen (DO) concentration at 2–4 mg/L. Between the aerator and the cathode, a polyvinylidene fluoride (PVDF) hollow-fiber curtain membrane module was placed, with a total membrane area of 0.14 m^2^ and a pore size of 0.04 μm. A pressure sensor was installed on the outlet pipe between the membrane module and the effluent peristaltic pump, connected to a paperless recorder for online monitoring and recording of the transmembrane pressure (TMP). Worms attached and grew on the porous carbon felt anode at a density of 0.42 g/cm^2^ (wet weight), corresponding to a dry weight density of 0.066 g/cm^2^. During operation, a portion of the sludge settled and covered the anode surface. The sludge retained on the anode served as nutrient source for the worms. A stirring magneton was placed at the center of the anode surface and rotated at 500 rpm for 5 min every 24 h, driven by a magnetic stirrer. This operation helped refresh the sludge after worm predation and efficiently remove worm excretions from the anode surface, thereby maintaining an optimal feeding environment for worm growth. A conventional MBR system (C-MBR) without worms or an MFC component was operated in parallel as a control system, and the configuration of the C-MBR was shown in Figure 1b. For each configuration (C-MBR and W-MBER), one reactor was operated due to the integrated and complex nature of the systems. To ensure statistical reliability, system performance were monitored over an extended and stable operational period. Moreover, all critical water quality and sludge property parameters were determined in triplicate independent samples collected from each reactor. The mixed liquor suspended solids (MLSS) in both the W-MBER and the C-MBR were maintained around 6000 mg/L through daily sludge wasting to ensure comparable operating conditions. The variations in MLSS and mixed liquor volatile suspended solids (MLVSS) of the W-MBER and C-MBR during the operation were illustrated in Figures S1 and S2.

#### 2.1.3. Worm Acclimation and Screening

The sludge predator *Limnodrilus hoffmeisteri* (Tubificidae) worms purchased from a flower and bird market in Baoding. Prior to use, the worms were washed to remove adhering sand and inactive individuals, and then acclimated for 15 days in basins containing activated sludge mixed with dechlorinated tap water. The water depth was maintained at 5 cm under shaded conditions, with oxygen supplied via microporous diffusers connected with an air-compression pump to sustain a dissolved oxygen (DO) level of approximately 1 mg/L. The sludge was replaced twice daily. Before inoculation, the worms were enveloped in filter cloth, rinsed, and distributed in a shallow tray. The tray was covered with gauze, submerged under 2–3 cm of water, and left undisturbed for 1 h under low-light conditions to allow the active worms to naturally emerge through the gauze. By lifting the corners of the gauze, the active worms were separated from the sludge. Excess surface water was gently absorbed using paper towels before weighing.

### 2.2. Inoculation and Operation Conditions

For reactor inoculation, the worms were transferred into the W-MBER tank with water, gently agitated to disperse clumps, and evenly distributed onto the carbon felt anode at the reactor bottom. Owing to their inherent benthic and thigmotactic behavior, the worms actively sought and colonized the three-dimensional porous matrix of the carbon felt, thereby establishing it as their stable primary habitat throughout the operation. Following the distribution of worms on the anode, the liquid was drained, and the reactor was filled with a mixture of activated sludge and synthetic wastewater, adjusted to a settled sludge height of 3 cm to fully cover the anode. The cathode was floated on the surface, and the electrodes were connected via an external resistor of 1000 Ω. The medium was replaced when the output voltage dropped below 50 mV. The successful formation of electrogenic biofilm on the anode was confirmed by reproducible maximum voltages over consecutive cycles. After the inoculation was completed, the reactor was drained, and the membrane module, aerator, and stirring magneton were installed. Subsequently, the reactor was filled with aerobic activated sludge at a concentration of 6000 mg/L. Meanwhile, the external resistance was switched from 1000 Ω to an operational resistance of 250 Ω. This adjustment aimed to promote higher current generation and enhance the associated bioelectrochemical effects while maintaining system stability by avoiding pseudo-short circuit conditions. The system was then switched to continuous-flow mode, with synthetic wastewater fed at a rate of 12.5 mL/min, resulting in a hydraulic retention time (HRT) of 6 h. The membrane module was operated at a constant flux of 5.4 L/(m^2^ h) with an intermittent suction cycle of 8 min on and 2 min off. Membrane fouling was monitored by tracking the transmembrane pressure (TMP). Chemical cleaning was performed once the TMP exceeded 30 kPa, which involved removing the membrane, rinsing it with tap water, soaking it in a 0.5% sodium hypochlorite solution for 2 h, and finally rinsing it thoroughly with tap water. The operational timeline and conditions for worm acclimatization, reactor inoculation, and system operation phases were shown in Table S1.

### 2.3. Analytical Methods

#### 2.3.1. Water Quality Analysis and Sludge Characterization

The Chemical oxygen demand (COD), ammonia nitrogen (NH_4_^+^-N), total nitrogen (TN), mixed liquor suspended solids (MLSS) and mixed liquor volatile suspended solids (MLVSS) were measured in three replicates based on the standard methods [15]. The SMPs and EPSs contents of the sludge in the reactors were obtained using previously reported procedures [16]. The concentrations of protein and carbohydrates in SMPs and EPSs were determined in three replicates by colorimetric method [17]. The particle size distribution of sludge flocs was observed using a particle size analyzer (Mastersizer 2000, Malvern, UK). The zeta potential was measured in three replicates by a Zetasizer Nano ZS Instrument (Malvern Instruments Ltd., Malvern, UK) at 25 °C. The contact angle was measured by a contact angle meter (SL150, KINO Industry, Boston, MA, USA), and each contact angle value was based on the arithmetic mean of at least twelve independent measurements.

#### 2.3.2. Electrochemical Analysis

The voltage across the resistance was monitored every 1 min using a paperless recorder (Pangu, Hangzhou, China). The polarization curves of the MFC were obtained by varying the external resistance from 5000 to 50 Ω. The maximum power density was determined from the polarization curves.

#### 2.3.3. Calculation of Sludge Production Rate and Sludge Yield

The concentrations of MLSS and MLVSS in C-MBR and W-MBER were measured every 2 days (Figures S1 and S2). The sludge yield coefficient of the C-MBR and W-MBER can be expressed as follows:

(1) $$Y\;=\;\frac{\left({\mathrm{MLVSS}}_{41}\;-{\;\mathrm{MLVSS}}_{1}\right)\;\times \;V_{\mathrm{MBR}}\;+\;\sum_{j=1}^{41}\left({\mathrm{MLVSS}}_{j}\;\times {\;V}_{j}\right)}{\sum_{j=1}^{41}\left(C_{Ij}\;-\;C_{Ej}\right)}$$

(2) $$Sludge\;production\;rate=\frac{\left({\mathrm{MLVSS}}_{41}\;-\;{\mathrm{MLVSS}}_{1}\right)\;\times \;V_{\mathrm{MBR}}+\sum_{j=1}^{41}\left({\mathrm{MLVSS}}_{j}\;\times {\;V}_{j}\right)}{41}$$

where Y was the sludge yield, kg VSS/kg COD_removed_; V_MBR_ was the volume of MBR, L; V*_j_* was the discharged sludge amount of the C-MBR and W-MBER on the day *j*, L; C_I*j*_ was the COD in the influent of the C-MBR and W-MBER on the day *j*, respectively, kg/d; C_E*j*_ was the COD in the effluent of the C-MBR and W-MBER on the day *j*, kg/d.

#### 2.3.4. Surface Energy Parameters

In order to analyze the effects of sludge properties on membrane fouling. The surface tensions of SMPs, EPSs, and sludge flocs were calculated based on the contact angle data according to the augmented Young-Laplace equation [18]:

(3) $$\gamma^{\mathrm{TOT}}=\gamma^{\mathrm{LW}}+\gamma^{\mathrm{AB}},$$

where *γ*^TOT^ is the total surface tension, mJ/m^2^; *γ*^LW^ is Lifshitz-van der Waals surface tension, mJ/m^2^. *γ*^AB^ is Lewis acid-base surface tension, mJ/m^2^. The detailed calculations for the terms in the surface thermodynamics analysis are shown in the Supporting Information.

#### 2.3.5. The Interaction Energy Calculation

The total interaction energy ($U_{\mathrm{mws}}^{\mathrm{XDLVO}}$) was calculated by Extended Derjaguin-Landau-Verwey-Overbeek (XDLVO) theory. According to this theory, the total interaction energy is obtained through the summation of AB interaction energy ($U_{\mathrm{mws}}^{\mathrm{AB}}$), LW interaction energy ($U_{\mathrm{mws}}^{\mathrm{LW}}$) and electric double layer (EL) interaction energy ($U_{\mathrm{mws}}^{\mathrm{EL}}$):

(4) $$U_{\mathrm{mws}}^{\mathrm{XDLVO}}\;=\;U_{\mathrm{mws}}^{\mathrm{AB}}\;+\;U_{\mathrm{mws}}^{\mathrm{LW}}\;+\;U_{\mathrm{mws}}^{\mathrm{EL}}$$

where the subscripts m, w and s correspond to the membrane, water and SMPs/EPSs/sludge flocs, respectively. The detailed calculations of the free energy of cohesion and adhesion and the total energies which are expressed as a function of the separation distance h are described in the Supporting Information.

## 3. Results and Discussion

### 3.1. Electricity Generation Performance of W-MBER System

**Figure 2:**
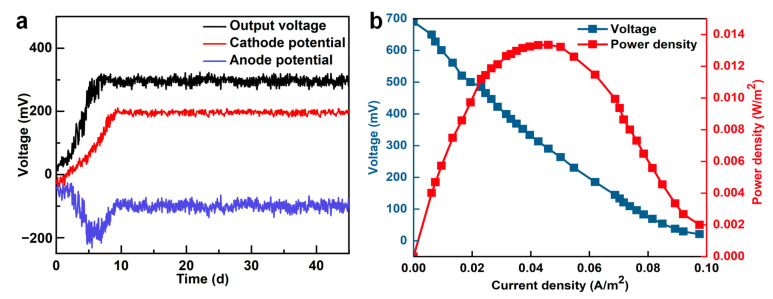
Variation in (**a**) voltage output with time and (**b**) power density and polarization curves of W-MBER.

As shown in Figure 2a, the W-MBER exhibited sustained and efficient electricity generation over the 41-day continuous-flow operation. After an initial 10-day period, the system reached a steady-state, maintaining a stable voltage output around 290 mV. During the first 10 days, the output voltage and cathode potential exhibited a similar variation trend, after which the cathode potential stabilized at approximately 190 mV. In contrast, the anode potential gradually decreased and stabilized at around −100 mV. Pt-C catalyst was added to the cathode, which made the reduction reaction of the cathode have high activation efficiency.

To determine the maximum power density and internal resistance, polarization and power density curves were measured during the stable operational phase of the W-MBER. As illustrated in Figure 2b, the W-MBER achieved an open circuit voltage of 690 mV and a maximum power density of 0.013 W/m^2^, respectively. The voltage and power outputs obtained in this system were comparable to those of other microbial fuel cells coupled with aerobic activated sludge systems. The internal resistance, derived from the slope of the polarization curve, was calculated as 346.27 Ω, which is notably lower than that of other integrated MBR configurations. It is worth noting that there were aerators at the bottom of the reactor between the anode and cathode to supply enough dissolved oxygen and ensure the active sludge was well-mixed. This turbulence likely enhanced proton (H^+^) transport from the anode to the cathode and improved oxygen transfer from the bulk liquid to the cathode biofilm. The electroactive bacteria in the outer layer of the cathode biofilm could transfer the electrons from the cathode and reduce the oxygen. Consequently, the turbulence between the electrodes contributed to a reduction in internal resistance, enhancing electricity generation efficiency in the W-MBER system.

### 3.2. Wastewater Treatment Performance

**Figure 3:**
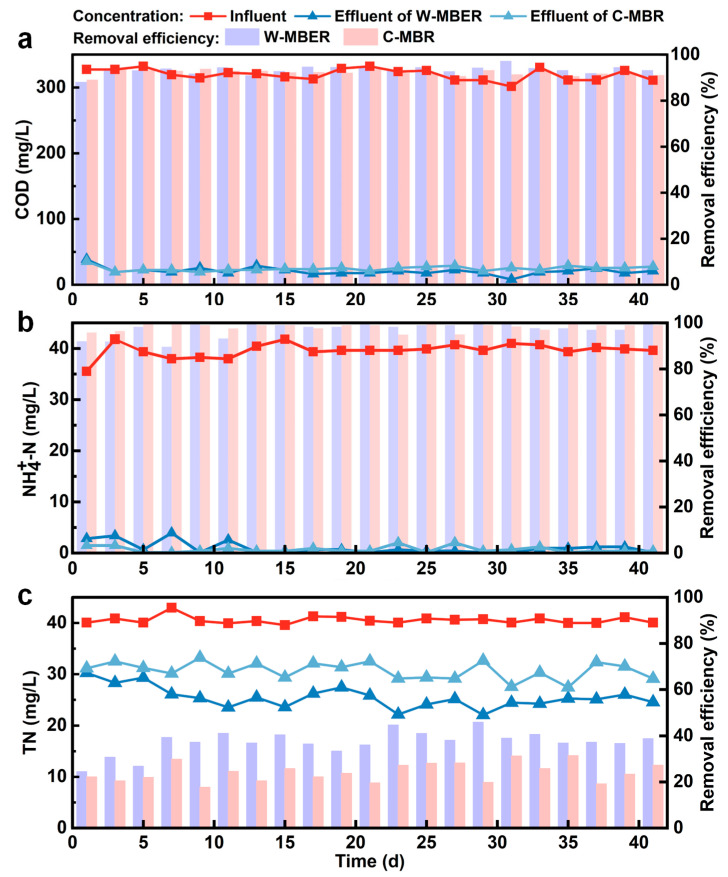
Concentrations and removal efficiencies of (**a**) COD, (**b**) NH_4_^+^-N and (**c**) TN in W-MBER and C-MBR.

**Figure 4:**
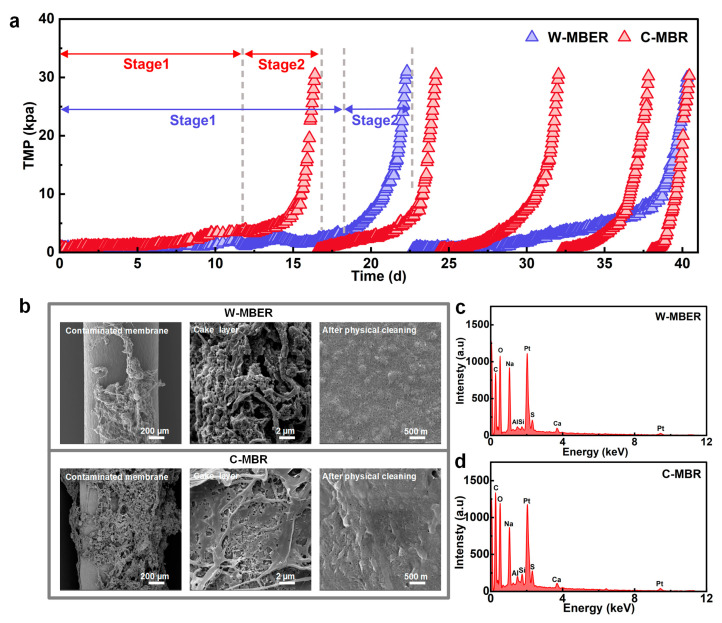
Variations in (**a**) TMP in the W-MBER and C-MBR. (**b**) SEM images of contaminated membrane, cake layer structure, and membrane surface after physical cleaning of the W-MBER and C-MBR. SEM-EDX analysis of foulants on membrane surface in the (**c**) MFC-MBR and (**d**) C-MBR.

The wastewater treatment performance of the W-MBER and C-MBR systems was evaluated based on the concentration and removal efficiency of COD, NH_4_^+^-N, and TN throughout the operational period (Figure 3). During the 41-day operation, COD concentration in both reactors decreased gradually and stabilized, with both systems demonstrating excellent organic contaminant degradation. The W-MBER achieved a slightly higher COD removal efficiency of 93.57%, compared to 92.57% in the C-MBR (Table S1). The enhanced COD removal in the W-MBER can be attributed to the integration of the microbial fuel cell (MFC), which stimulated microbial activity via bioelectrochemical processes [19]. In this system, exoelectrogenic bacteria oxidize organic substrates through extracellular electron transfer (EET) to the anode, a mechanism supported by the significantly increased specific oxygen uptake rate (SOUR) observed in the W-MBER (Figure 4c). This bioelectrochemically driven metabolism not only contributed to current generation but also promoted a more active and robust microbial community, thereby improving organic degradation efficiency [20]. While some studies suggest that worm predation may lead to the release of organic matter [2,21,22], the integration of an MFC in W-MBER introduced electroactive bacteria whose activity appears to counteract this effect. The W-MBER not only avoided a decline in performance but actually achieved higher COD removal. This indirectly confirms that the microbial community’s overall activity was enhanced. Thus, the MFC component creates a niche that enriches exoelectrogens. Their EET-based metabolism not only generates current but also contributes to a more robust and active microbial community for organic degradation. Furthermore, the reported research has indicated a positive correlation between COD concentration and membrane fouling propensity [23]. Higher organic loading typically promotes the formation and thickening of a biofilm layer on the membrane surface. Therefore, the reduction in COD concentration observed in the W-MBER likely contributes to the alleviation of membrane fouling.

The W-MBER also demonstrated considerable TN and NH_4_^+^-N removal efficiency. As shown in Figure 3b, the ammonia nitrogen removal efficiencies of W-MBER and C-MBR systems were 98.61% and 98.45%, respectively. The supernatant from W-MBER system exhibited 0.78 mg/L residual ammonia nitrogen, representing an 18.13% reduction compared to the C-MBR. An anaerobic environment was generated at the bottom of the reactor. Under anaerobic environments, dead worms and microbial decay in activated sludge releases ammonium, while ammonifying bacteria mediate organic nitrogen conversion. Furthermore, worm predation processes introduced metabolites into the sludge matrix, significantly elevating ammonium concentrations. The experimental results showed that, the W-MBER system still had good ammonia nitrogen removal performance.

The average TN removal rates for the W-MBER and C-MBR were 37.24% and 24.31%, respectively, representing a 12.93% improvement in the W-MBER (Figure 3c). This enhancement indicates that the combined introduction of a micro-electric field and worm predation can effectively improve TN removal in an MBR. Aerobic sludge absorbed a certain amount of nitrogen to promote its own growth, which was the main factor causing the denitrification of aerobic sludge. With the extension of operation time, a large amount of sludge accumulates inside the worm filler, and the dissolved oxygen inside and outside the filler was quite different. The diversified aerobic and anaerobic environment causes denitrification in the W-MBER system. In addition, the worm predation on the sludge and the peristaltic of worms would accelerate the material exchange between the mud and water. Thus, the removal of total nitrogen in the experimental system was superior to that in the control system [24,25]. In addition, there was a carbon fiber cathode in the W-MBER system. During operation, the sludge adhered to the carbon cloth of the cathode to form a layer of biofilm. When the biofilm thickened, due to the blockage of oxygen mass transfer, anaerobic denitrifying bacteria was generated inside the biofilm, and the denitrifying bacteria used the organic matter in the sludge to reduce nitrate ions to nitrogen to achieve nitrogen removal [26,27].

### 3.3. Membrane Fouling Mitigation in the Combined System

Under constant flux operation, TMP increases with rising membrane resistance; therefore, the TMP growth curve directly reflects the trend of membrane fouling. The TMP variations in W-MBER and C-MBR were continuously monitored, as shown in Figure 4a. In the process of operation, when the TMP was higher than 30 kPa, the membrane module was carried out. After physical cleaning with pure water, the cleaned membrane was loaded into the system again and entered the next membrane filtration cycle.

Over the 41-day operation, the membrane in the W-MBER system underwent physical cleaning once and completed two filtration cycles, whereas the C-MBR system required four physical cleanings and completed five cycles. This demonstrates that the W-MBER system achieved a longer operational cycle and reduced the frequency of membrane cleaning. The initial filtration cycle lasted 23 d for the W-MBER and 16 d for the C-MBR, respectively. The average TMP growth rate of W-MBER system was 1.3 kPa/d, which was 31.58% lower than that of C-MBR system (1.9 kPa/d), indicating significantly mitigated membrane fouling in the W-MBER. According to Ognier et al. [28], membrane fouling in an MBR can be divided into two distinct stages: a stable TMP growth stage followed by a TMP jump stage. The increase in TMP is mainly due to the blockage of membrane pores and the gradual formation of mud cake layer, while the rapid growth of TMP is due to the formation of a dense mud cake layer on the membrane surface, resulting in a sharp increase in membrane resistance and a sudden drop in membrane flux [29]. The operation time of the first and second stages of membrane pollution in the W-MBER system were 18 days and 5 days, while those of the C-MBR system were 12 days and 4 days. The electric field force in W-MBER would drive the negative membrane pollutants away from the MBR membrane, reducing the probability of pollutants and membrane contact. Secondly, under the effect of low electric field stimulation and worm predation, the physiological characteristics of microorganisms will be affected, resulting in a series of changes in sludge properties, thus easing the membrane pollution rate [30].

To quantitatively assess membrane fouling characteristics, we analyzed the membrane resistance distribution and growth rates for the W-MBER (day 23) and C-MBR (day 16) systems when transmembrane pressure exceeded 30 kPa (Table S3). W-MBER demonstrated a 42.31% reduction in total resistance growth rate compared to the C-MBR system. Cake layer resistance (R_c_) dominated total resistance in both systems, accounting for 89.55% in W-MBER and 91.38% in C-MBR. The W-MBER system exhibited substantially lower cake layer resistance and a 43.51% reduction in average R_c_ growth rate compared to C-MBR. These results indicate that the synergistic effects of the electric field and worms significantly retard cake layer formation. The mechanism likely involves reduced adhesion strength between foulants and the membrane surface, where back-transport forces exceed adhesion forces according to established force balance equations [31]. The W-MBER system showed 10.65% higher cumulative pore fouling resistance (R_f_ = 10.18 × 10^11^ m^−1^) than the C-MBR system (9.20 × 10^11^ m^−1^). However, the average R_f_ growth rate was 24.13% lower in W-MBER. The higher cumulative resistance reflects longer operational cycles in W-MBER, providing more opportunities for pore blocking. Conversely, the reduced growth rate indicates slower pore fouling kinetics, likely due to altered sludge properties that minimize critical membrane fouling conditions. These resistance analyses confirm that the combined electric field and worm effects effectively mitigate both cake layer and pore fouling. Since soluble microbial products primarily drive pore blocking while extracellular polymeric substances largely determine cake layer resistance [32,33].

In the biological fragmentation pathway, predation involving worm feeding, fragmentation, and digestion changes the structure of cake layer (Figure 4b–d and Figure S3). Scanning electron microscopy (SEM) revealed that the fouled membrane in the W-MBER exhibited fewer and more loosely aggregated cake layer compared to the C-MBR. This resulted in the clear membrane pores being visible on the W-MBER membrane after physical cleaning, while a dense fouling layer was still present on the C-MBR membrane.

### 3.4. Analysis of Sludge Characteristics

#### 3.4.1. Analysis of Sludge Activity

**Figure 5:**
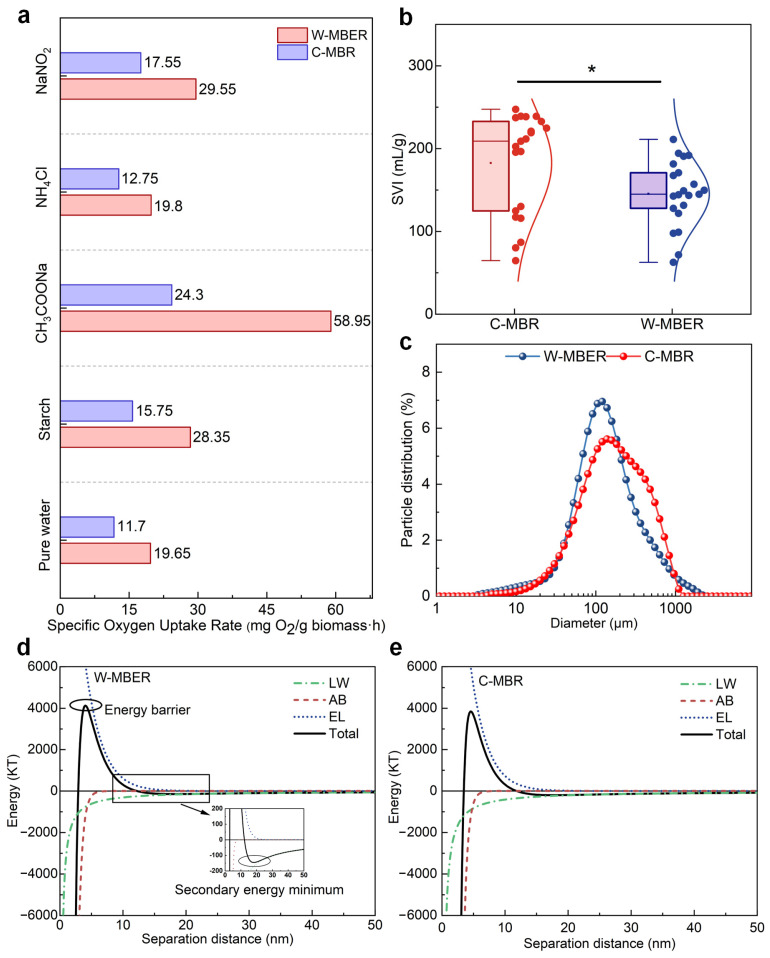
(**a**) SOUR of the sludge in the W-MBER and C-MBR under different substrates. (**b**) Sludge volume index (SVI) distribution of the W-MBER and C-MBR during the entire operation (*, *p* < 0.05). (**c**) Size distribution of the sludge flocs in the W-MBER and C-MBR. (**d**,**e**) interaction energy curves of the sludge flocs in the W-MBER and C-MBR.

To evaluate the combined effects of electric field exposure and worm predation on sludge activity, SOUR measurements were performed using multiple substrate conditions. Five different substrates were tested: pure water, starch (500 mg/L), sodium acetate (CH_3_COONa, 641 mg/L), ammonium chloride (NH_4_Cl, 20 mg/L), and sodium nitrite (NaNO_2_, 20 mg/L). The corresponding results are presented in Figure 5a.

In the pure-water baseline, which supplied no external nutrients, oxygen consumption reflected endogenous respiration. The W-MBER system demonstrated a higher endogenous respiration rate compared to the C-MBR system, indicating enhanced overall sludge activity. Organic substrate degradation capacity was assessed using starch and sodium acetate, with oxygen consumption rates reflecting COD removal efficiency. Compared to the C-MBR, the W-MBER exhibited 44.44% and 58.78% higher oxygen consumption rates for starch and sodium acetate, respectively, indicating that the synergistic effects of electric field application and worm activity significantly enhance the sludge’s organic matter degradation capacity.

Nitrogen transformation processes were evaluated using ammonium chloride and sodium nitrite as substrates, where oxygen consumption rates indicated nitrification activity. Compared to C-MBR system, the W-MBER system achieved 55.29% and 15.78% higher ammonia oxidation rates for the two substrates, respectively, confirming that the integrated treatment strategy effectively improves nitrogen removal performance.

#### 3.4.2. Analysis of Sludge Reduction

To evaluate the sludge reduction performance of W-MBER system, the sludge yield coefficient and sludge production rate of the two systems was analyzed. The calculated results were shown in Table 1. The sludge yield coefficient of the W-MBER and C-MBR systems were reduced by 72.00% and 62.00%, respectively, compared with the conventional activated sludge process (0.50 kgVSS/kgCOD removed). The microorganisms in MBR system were in a high endogenous respiratory state; so, they were regarded as having the ability to reduce sludge.

Furthermore, compared with C-MBR system, the sludge production in W-MBER system was reduced by 28.51%. This enhancement in sludge reduction efficiency was likely due to anodic electricity generation and worm predation in addition to sludge anaerobic digestion at the bottom of W-MBER system. However, Luxmy et al. [34] reported that the presence or absence of the metazoan population had no significant effects on the sludge yield in the MBRs, even when the metazoan population reached up to 1000–2000 per mL mixed liquor. The aquatic worms exhibited a marked discrepancy in sludge reduction efficiency between the present study and the study of the other researchers. It was hypothesized that the variation in sludge reduction efficiency observed between the present study and the aforementioned study might be attributable to differences in aquatic worm species. Another possible reason is the different growth status of the worms. In this study, the worms were attached to the filler surface, whereas in their study, the metazoans were attached to the membrane surface. In addition, the stimulation of electric field in W-MBER system leads to the increase in the endogenous respiration rate of activated sludge, which will also improve the sludge reduction effect of W-MBER system.

#### 3.4.3. Analysis of Sludge Settleability

The changes in the sludge volume index (SVI) in the W-MBER and C-MBR systems were shown in Figure 5b and Figure S3. In both systems, SVI values showed an overall increasing trend, indicating a gradual decline in sludge settleability over time. The sludge settling characteristics, as measured by SVI, differed significantly between the two reactor systems (*p* < 0.05). The C-MBR system exhibited SVI values ranging from 60 to 250 mL/g with a median of approximately 210 mL/g. In contrast, the W-MBER system showed substantially stabled settling behavior, with SVI values between 70 and 210 mL/g and a median of 145 mL/g, representing a 31% reduction compared to the C-MBR. The distribution pattern reveals that the W-MBER system not only achieved lower median SVI values but also demonstrated reduced variability, as evidenced by the smaller interquartile range. This suggests more consistent sludge settling performance throughout the operational period. The improved settling characteristics likely result from selective predation by aquatic worms on dispersed bacteria and fine particulates that typically contribute to poor settling properties. This predation activity promotes the formation of larger, denser bio-flocs with enhanced settling capabilities. The reduced variability in SVI values for the W-MBER system suggests that worm predation provides a biological buffering mechanism that maintains consistent sludge quality despite fluctuations in operational conditions.

#### 3.4.4. Analysis of Sludge Aggregation Ability

Figure 5c shows that the size of the sludge flocs in the W-MBER system was smaller in size but more uniform than those in the C-MBR system. This structural change is attributed to worm predation, which significantly reduced the abundance of filamentous bacteria (Figure S4) and promoted physical fragmentation of the sludge, resulting in smaller particle sizes [35]. Aggregation analysis further indicated that the energy barrier for sludge particles in the W-MBER system was 4131.66 KT, 7.45% higher than that in the C-MBR system (3845.11 KT), suggesting that the sludge in the W-MBER system is less prone to aggregation. Correspondingly, the secondary energy minimum value was 28% lower in the W-MBER system, indicating less stable sludge aggregates compared to the C-MBR.

Although such fragmentation and reduced aggregation could theoretically increase the risk of membrane fouling due to higher concentrations of fine particles, no severe fouling was observed during the operation. This may be attributed to the counteractive effect of the integrated microbial fuel cell, which enhances microbial activity and organic turnover, thereby mitigating potential negative impacts. Nevertheless, the long-term implications of sustained worm predation on sludge morphology and system stability warrant further investigation.

### 3.5. Mechanisms of Membrane Fouling Mitigation

#### 3.5.1. Concentrations of SMPs and EPSs

**Figure 6:**
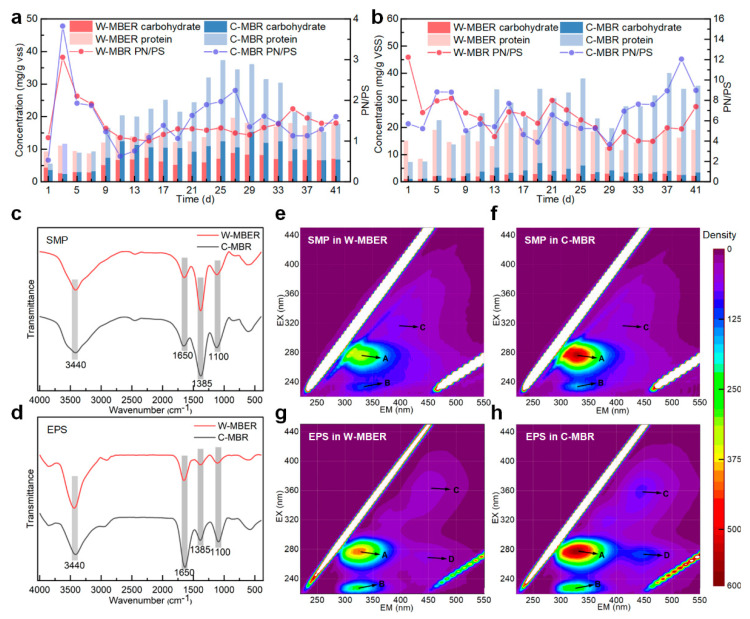
Variations in the (**a**) SMPs and (**b**) EPSs concentrations in the W-MBER and C-MBR. FTIR spectra of (**c**) SMPs and (**d**) EPSs in the W-MBER and C-MBR. EEM spectra of (**e**,**f**) SMPs and (**g**,**h**) EPSs in the W-MBER and C-MBR. (Peak A: tryptophan protein-like substances; Peak B: tyrosine protein-like substances; Peak C: humic acid-like substances; Peak D: fulvic acid-like substances).

SMPs are organic substances produced and released into supernatant by microorganisms in the process of substrate metabolism and their own decay, which are mainly composed of polysaccharides and proteins [36]. The majority of soluble organic matter in sludge supernatant is SMPs [37]. A large number of studies had proved that the concentration, composition and characteristics of SMPs in MBR were closely related to membrane fouling. Figure 6a demonstrates that SMPs concentration in the W-MBER system remained relatively stable throughout operation, peaking at day 20. The W-MBER system achieved a significantly lower average SMPs concentration of 14.50 ± 3.24 mg/L compared to 22.01 ± 8.92 mg/L in the C-MBR system (*p* = 0.0016), representing a 34.12% reduction. The dual stimulation effects of micro-electric fields and worm predation enhanced aerobic sludge activity, improving the degradation of organic compounds in the sludge supernatant and further reducing SMPs content. Specifically, protein (PN) and polysaccharide (PS) concentrations in W-MBER-derived SMPs decreased by 35.17% and 32.82%, respectively, compared to C-MBR values. The higher degradation rate observed for polysaccharides reflects their inherently greater biodegradability relative to proteins. Since SMPs represents a primary contributor to membrane fouling in MBR systems, reducing SMPs concentration directly benefits membrane performance. The protein-to-polysaccharide ratio (PN/PS) also influences fouling behavior, with the W-MBER system achieving a slightly lower PN/PS value of 1.36 compared to 1.41 in the C-MBR system (3.55% reduction).

EPSs are organic polymers released during microbial substrate degradation and cell lysis, forming protective matrices around bacterial cells. These biopolymers can also incorporate pre-existing organic matter from wastewater, generating complex structures that significantly influence microbial metabolism and sludge properties. EPSs directly affects critical operational parameters including floc morphology, surface charge distribution, flocculation efficiency, and dewatering properties [38,39]. Therefore, both the content and composition of EPSs are critical determinants of membrane fouling in MBR systems. Operational data revealed distinct EPSs dynamics between the two reactor configurations (Figure 6b). In the W-MBER system, EPSs levels remained stable during the first 20 days and then showed a consistent decline over the subsequent 20 days. In contrast, the C-MBR system exhibited a consistent upward trend throughout the operational cycle. This substantial decrease demonstrates that the synergistic effects of electric field stimulation and worm predation effectively suppress EPSs accumulation. The enhanced EPSs degradation in the W-MBER system can be attributed to improved microbial utilization of these polymers as alternative carbon sources. Compositional analysis revealed that protein and polysaccharide concentrations in the W-MBER system decreased to 14.56 mg/gVSS and 2.42 mg/gVSS, respectively—representing reductions of 36.42% and 33.33% compared to the C-MBR system. The preferential reduction in protein content decreased positively charged amino groups while relatively increasing negatively charged carboxylic groups. This shift enhanced the absolute zeta potential of sludge particles and intensified electrostatic repulsion between the negatively charged sludge and membrane surfaces [40]. The resulting inhibition of particle deposition and cake layer formation contributed significantly to reduced membrane fouling.

#### 3.5.2. FTIR Analysis of SMPs and EPSs

Fourier-transform infrared (FTIR) spectroscopy was employed to characterize the major functional groups in the SMPs and EPSs extracted from the W-MBER and C-MBR systems. As demonstrated in Figure 6c, the peak near 1660 cm^−1^ corresponds to the amido-|(C=O), indicating the presence of proteins [41]. The broad peak centered at 1100 cm^−1^ and attributed to carbon-oxygen bonds was identified as being indicative of carbohydrates or carbohydrates-like substances [42]. In the W-MBER system, the peak intensities for both protein and carbohydrate regions significantly decreased. Figure 6d presents the FTIR spectra of the EPSs in the two reactors. In both systems, EPSs had absorption peaks at 1650, 1385, and 1100 cm^−1^, indicating that each system’s EPSs contained proteins, humic acid, and polysaccharides. Compared with the C-MBR system, the absorption peak intensity at 1653 cm^−1^ in the W-MBER system decreased, which was due to the reduction in C=O functional groups, suggesting a decrease in protein content. The absorption peak intensity at 1100 cm^−1^ in the W-MBER system was also lower than that of the control system, indicating a reduction in the number of functional groups related to polysaccharides and a decrease in polysaccharide content. The absorption peak intensity at 1385 cm^−1^ in the W-MBER system was even lower, suggesting a decrease in the content of humic acid-like substances in the system. The above analysis results indicate that compared with the C-MBR system, the absorption peak intensities of protein, polysaccharides, and humic acid-like substances in the W-MBER system were weaken, indicating that the synergistic effect of the electric field and worms in the W-MBER system can reduce the content of pollutants and alleviate membrane fouling.

#### 3.5.3. Fluorescence Characteristics Analysis of SMPs and EPS

Figure 6e–h shows the Three-dimensional excitation-emission matrix (EEM) fluorescence spectra of SMPs and EPSs in the W-MBER and C-MBR systems. Four main peaks (Peak A, B, C and D) were readily identified, corresponding to tryptophan protein-like substances, tyrosine protein-like substances, humic acid and fulvic acid, respectively. The fluorescence intensity of Peak A (tryptophan-like) in the SMPs of the W-MBER was 27.79% lower than that in the C-MBR, while Peak B (tyrosine-like) intensity decreased by 13.71%, indicating reduced protein content in the W-MBER. In addition, the fluorescence peak intensity of humic acid (Peak C) and fulvic acid (Peak D) were also lower in the W-MBER, suggesting a decrease in these substances, likely associated with the concomitant degradation of proteins. The location and intensity of fluorescence peaks of SMPs and EPSs in W-MBER and C-MBR systems are shown in Table S4. It could be seen that the fluorescence intensity of tryptophan proteins in the W-MBER system was 36.49% lower than that of C-MBR, and tyrosine proteins decreased by 30.86%. These reductions demonstrated that the synergistic action of the electric field with the worm predation can degrade these proteins in the W-MBER system. In contrast, humic acid fluorescence changed only slightly between the two systems and remained at low levels, implying that humic acids could pass more readily through the membrane pores with the effluent. However, protein typically have larger molecular weights and are less permeable, making them more prone to cause membrane pore blockage. As reported by Wang et al. [43], higher protein concentrations correlate with more severe membrane fouling, and fouling rate is negatively correlated with protein fluorescence intensity. Therefore, reducing protein content can slow down the occurrence of membrane contamination.

The locations of Peak B in the EPSs of the C-MBR and W-MBER exhibited a 4 nm blue shift towards shorter wavelengths along the emission axis. This shift was attributed to the impact of worm predation. The two main peaks (Peak A and B) related to protein-like substances manifested within the EEM fluorescence spectra of membrane foulants [44]. Wietlik et al. [45] confirmed that blue-shift was ascribed to the elimination of particular functional groups such as carbonyl, hydroxyl and amine, a reduction in the degree of p-electron systems, and the decrease in the number of aromatic rings and conjugated bonds in a chain structure. Li et al. [46] a decrease in the protein-like substances associated with Peak B in EPSs contributes to reduced membrane fouling. Therefore, the reduced content and structural changes in these protein-like substances in the EPSs likely play a key role in mitigating membrane fouling in W-MBER. It could be seen that the protein components in EPSs were significantly reduced after electric field stimulation and worm predation in W-MBER system. Additionally, the skeleton structure was simpler, and membrane fouling was lighter. These results suggested that worm predation reduced the number of unsaturated groups. The reduction in these electronegative groups effectively reduces the Zeta potential of SMPs and EPSs.

#### 3.5.4. Interaction Energy Between Membrane Surface and Foulants

SMPs, EPS, and sludge flocs constitute the primary foulants that readily adhere to and accumulate on the membrane surface, forming gel or cake layers which result in flux decline during filtration. This fouling process is governed by the surface properties of the foulants, such as hydrophobicity and surface charge, and their interaction energies with the membrane. These factors can be quantitatively analyzed by the XDLVO theory.

The surface energy parameters of PVDF membrane, SMPs, EPSs, and sludge flocs in W-MBER and C-MBR are presented in Table S5. The cohesion free energy (Δ*G*_coh_) presented in the table corresponds to the free energy per unit area between two identical materials in water, serving as a quantitative indicator of the hydrophobicity/hydrophilicity of the membrane and foulants [47,48,49]. A positive Δ*G*_coh_ value indicates a hydrophilic surface, whereas a negative value signifies a hydrophobic one. Therefore, the PVDF membrane exhibited hydrophilic properties (Δ*G*_coh_ > 0), whereas SMPs, EPSs, and sludge flocs in both systems showed strong hydrophobicity (Δ*G*_coh_ < 0). As shown in Table S6, the ΔG_adh_ values between SMPs and the membrane were negative in both systems, indicating an attractive interaction between the SMPs and the membrane surfaces. SMPs in the C-MBR showed stronger hydrophobicity, as evidenced by their higher Δ*G*_coh_ values compared to those in the W-MBER. thus SMPs in C-MBR was supposed to have higher interaction energy with the SMP-membrane at contact, which was confirmed by the higher value of ΔG_adh_. These results demonstrate that the adhesive free energy between SMPs and the membrane was reduced in the W-MBER, which could help mitigate SMPs adsorption and enhance membrane permeability.

**Figure 7:**
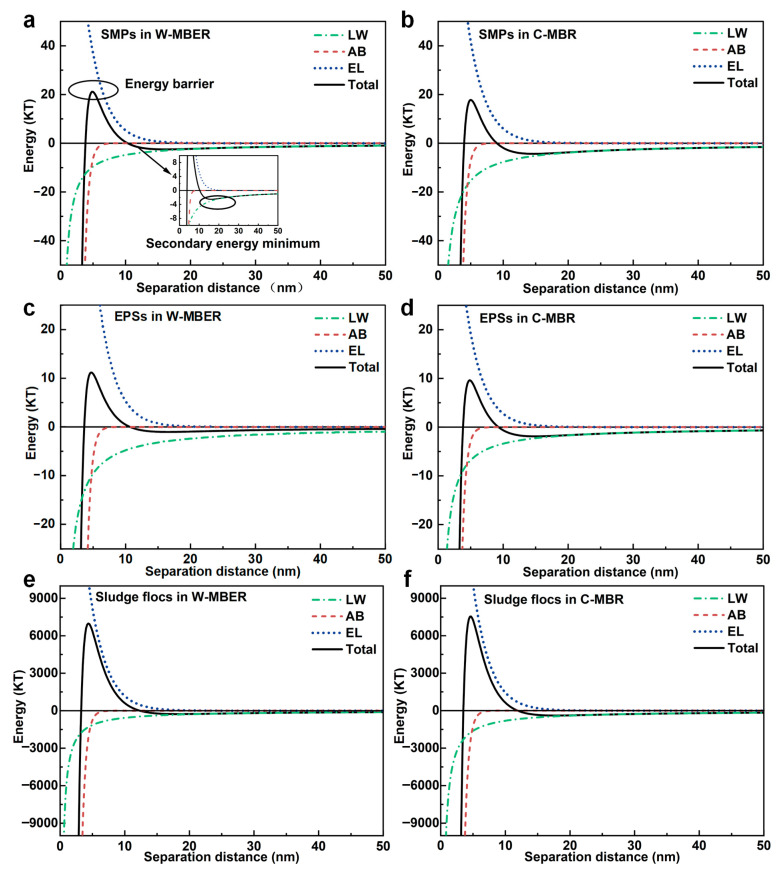
Curves showing variations in interaction energies between foulants (**a**,**b**) SMPs, (**c**,**d**) EPSs, and (**e**,**f**) sludge flocs and membrane in MFC-MBR and C-MBR with the separation distance.

The interaction energy profiles between SMPs or EPSs and the membrane as a function of separation distance were calculated using the XDLVO theory (Figure 7a,b). The interaction energy profiles revealed characteristic features that govern pollutant adhesion behavior. All systems exhibited an energy barrier that pollutants must overcome to reach the membrane surface, followed by a secondary energy minimum where particles become trapped. Higher energy barriers indicate greater difficulty for pollutants to approach and adhere to membranes, while deeper secondary energy minima require more energy for pollutant detachment. For SMPs, the interaction energy varied with distance. At 50 nm, weak attractive forces gradually strengthened as particles approached the membrane, reaching maximum attraction at approximately 13 nm (the secondary energy minimum). Below 10 nm, electrostatic repulsion dominated, creating an energy barrier with maximum repulsion at 5 nm. The W-MBER showed superior fouling resistance through modified energy relationships. SMPs in W-MBER faced a 21.09 KT energy barrier compared to 17.78 KT in C-MBR—a 1.2-fold increase that significantly impeded membrane adhesion. Additionally, the secondary energy minimum was notably lower in W-MBER, facilitating easier particle detachment under external forces such as crossflow or backwashing.

EPSs behavior followed similar patterns but with distinct characteristics. The attractive force peaked at 14 nm separation before transitioning to repulsive forces below 10 nm, with maximum repulsion occurring at 4 nm. The W-MBER system demonstrated even more pronounced anti-fouling effects for EPS, with a 16.65% higher energy barrier and a 46.03% lower secondary energy minimum compared to C-MBR. These changes indicate that the synergistic effects of worm predation and electric field stimulation in W-MBER systems fundamentally alter pollutant-membrane interactions. The increased energy barriers impede initial EPSs adhesion, while reduced secondary energy minima promote easier detachment, collectively minimizing membrane fouling propensity. The comprehensive energy analysis demonstrates that W-MBER systems create thermodynamically unfavorable conditions for membrane fouling through systematic modification of interfacial interactions, providing a mechanistic explanation for the observed performance improvements.

Sludge floc surface properties fundamentally determine membrane fouling behavior, with hydrophobicity and surface charge serving as primary controlling factors. Both systems produced hydrophobic sludge flocs, as indicated by negative cohesive free energy values (Table S5). However, W-MBER sludge demonstrated substantially reduced hydrophobicity (−21.95 mJ/m^2^) compared to C-MBR sludge (−43.51 mJ/m^2^), representing a 49.6% decrease in hydrophobic character. This hydrophobicity reduction stems directly from worm predation effects on microbial community structure. Long-term worm predation effectively suppressed filamentous bacteria populations, which are known to dramatically enhance sludge surface hydrophobicity. By preventing filamentous bacteria proliferation and associated sludge bulking, the W-MBER system fundamentally altered surface characteristics in ways that discourage membrane fouling. The thermodynamic driving force for sludge adhesion was substantially weakened in W-MBER systems. Adhesion free energy analysis revealed a 41.14% reduction compared to C-MBR systems, indicating significantly diminished affinity between sludge flocs and clean membrane surfaces (Table S6). Additionally, secondary energy minima, which determine particle detachment difficulty decreased substantially in W-MBER systems (Figure 7e,f). Sludge-to-membrane interactions showed a 32.32% reduction. This reduced energy minima facilitate easier sludge detachment under operational forces such as crossflow shear or backwashing.

The integrated energy analysis demonstrates that W-MBER systems maintain superior fouling resistance throughout the entire membrane operation cycle. The system effectively inhibits initial sludge adhesion to membrane surfaces while simultaneously promoting easier detachment from established cake layers. This dual-action mechanism retards sludge accumulation and prevents cake layer development, providing comprehensive fouling control. These findings establish that worm predation creates thermodynamically unfavorable conditions for membrane fouling through systematic modification of sludge surface properties. The combined effects of reduced hydrophobicity, weakened adhesion interactions, and enhanced desorption capacity collectively explain the superior performance observed in W-MBER systems.

## 4. Conclusions

Addressing the challenges of sludge reduction, energy recovery, and membrane fouling in MBR, this study developed a novel W-MBER system integrating aquatic worms and a microbial fuel cell into a conventional MBR. The W-MBER exhibited high wastewater treatment performance with removal efficiencies of 93.57% for COD, 98.61% for NH_4_^+^-N, and 37.24% for TN. Additionally, the system achieved a stable output voltage of 290 mV and a maximum power density of 0.013 W/m^2^, along with a 28.51% reduction in sludge production. In the W-MBER, the combined effect of worm predation and electrical stimulation enhanced the biological fragmentation and bacterial metabolism, thus improving the cake layer structure and membrane filtration performance. Moreover, thermodynamic analysis revealed that the energy barriers between both SMPs and EPSs and membrane surface increased by 18.61% and 16.65%, respectively, indicating a lower membrane fouling propensity. Overall, the novel W-MBER system developed in this study provides an effective technical solution for highly efficient sludge reduction, energy recovery and membrane fouling mitigation. Nevertheless, the long-term stability of the worm population in complex wastewater and the optimal configuration of key operational parameters, such as the external resistance, require further investigation. Future studies should focus on pilot-scale validation and dynamic control strategies to advance the practical application of the W-MBER technology.

## Figures and Tables

**Table 1:** Sludge yield and reduction efficiencies of W-MBER and C-MBR.

	Sludge Yield Coefficient (kg VSS/kg COD_removed_)	Sludge Production Rate (mg MLVSS/d)	Sludge Reduction Efficiency (%)
W-MBER	0.14	397.58	28.51%
C-MBR	0.19	556.12	—

## Data Availability

The original contributions presented in this study are included in the article/Supplementary Material. Further inquiries can be directed to the corresponding authors.

## Supplementary Materials

The following supporting information can be downloaded at: https://www.mdpi.com/article/10.3390/membranes16010002/s1, Figure S1: Variations in MLSS in the W-MBER and C-MBR; Figure S2: Variations in MLVSS in the W-MBER and C-MBR; Figure S3: Variations in SVI in W-MBER and C-MBR; Figure S4: Sludge flocs in W-MBER and C-MBR; Figure S5: Schematic diagram of mechanisms of fouling depression in W-MBER; Table S1. Operational timeline and conditions for worm acclimatization, reactor inoculation, and system operation phases; Table S2: Average concentration of COD, NH_4_^+^-N and TN in influent and effluent of W-MBER and C-MBR; Table S3: The membrane resistance and membrane resistance growth rate of W-MBER and C-MBR; Table S4: Fluorescence spectral parameters of the SMPs and EPSs in W-MBER and C-MBR; Table S5: Surface energy parameters (mJ/m^2^) of clean membrane and SMPs/EPSs/sludge flocs in W-MBER and C-MBR; Table S6: XDLVO predictions for interaction energy (mJ/m^2^) at contact between membrane and SMPs/EPSs/sludge flocs in W-MBER and C-MBR.
