# Supplementary material for: Performance of a Novel Worm-Assisted Membrane Bioelectrochemical System: Electricity Recovery, Sludge Reduction, and Membrane Fouling Mitigation
Source: Membranes (Basel). 2025 Dec 22;16(1):2. doi: 10.3390/membranes16010002 (PMC12844489; doi:10.3390/membranes16010002)
Supplement: Supplementary file 1 [file membranes-16-00002-s001.zip › membranes-4014951-supplementary.pdf]

membranes

Supplementary Materials

Performance of a Novel Worm-Assisted Membrane Bioelectrochemical System: Electricity Recovery, Sludge Reduction, and Membrane Fouling Mitigation

Chenyu Ding [1], Xin Guo [1], Weiye Bian [1], Zhipeng Li [2], Yang Li [1], Hongjie Wang [1,3,*] and Hui Li [1,3,*]

1 Hebei Key Laboratory of Close-to-Nature Restoration Technology of Wetlands, School of Eco-Environment, Hebei University, Baoding 071002, China
2 School of Marine Science and Technology, Harbin Institute of Technology at Weihai, Weihai 264209, China
3 Engineering Research Center of Ecological Safety and Conservation in Beijing-Tianjin-Hebei (Xiong'an New Area) of MOE, China
* Correspondence: wanghj@hbu.edu.cn (H.W.); lihuihbu@hbu.edu.cn (H.L.)

1. Surface energy parameters

The surface tension of sludge flocs and membrane was calculated based on the augmented Young-Laplace equation as follows.

$$(1+\cos\theta)\gamma_l^{TOT}=2\left(\sqrt{\gamma_s^{LW}\gamma_l^{LW}}+\sqrt{\gamma_s^{+}\gamma_l^{-}}-\sqrt{\gamma_s^{-}\gamma_l^{+}}\right) \quad (1)$$

$$\gamma^{AB}=2\sqrt{\gamma^{+}\gamma^{-}} \quad (2)$$

$$\gamma^{TOT}=\gamma^{LW}+\gamma^{AB} \quad (3)$$

Where θ is the contact angle, ∘. γ^{TOT} is the total surface tension, mJ/m^2. γ^{LW}, γ^{+} and γ^{-} are the components of Lifshitz-van der Waals, electron-acceptor and electron-donor, respectively, mJ/m^2. γ^{AB} is Lewis acid-base surface tension, mJ/m^2. *s* and *l* represent the solid and liquor samples. The contact angles of membrane, SMPs, EPSs, and sludge flocs with sterile water, diiodomethane and formamide were measured through a contact angle meter (SL150, American Kono Group, USA), respectively. The contact angle of each sample with each liquid was measured at least 10 times and the average value was adopted. The sludge flocs were sampled on day 80 of the operation.

2. Free energy of adhesion and cohesion

Surface tensions for membranes and colloids calculated using the acid-base approach can be used to evaluate the free energies of adhesion and cohesion per unit area between these surfaces.

The Lifshitz-van der Waals (LW), acid-base (AB) and electrostatic (EL) free energies per unit area are calculated according to the following equations.

$$\Delta G_{mws}^{LW}=2\left(\sqrt{\gamma_w^{LW}}-\sqrt{\gamma_m^{LW}}\right)\left(\sqrt{\gamma_s^{LW}}-\sqrt{\gamma_w^{LW}}\right) \quad (4)$$

$$\begin{aligned}\Delta G_{mws}^{AB} &=2\sqrt{\gamma_w^{+}}\left(\sqrt{\gamma_m^{-}}+\sqrt{\gamma_s^{-}}-\sqrt{\gamma_w^{-}}\right)+2\sqrt{\gamma_w^{-}}\left(\sqrt{\gamma_m^{+}}+\sqrt{\gamma_s^{+}}-\sqrt{\gamma_w^{+}}\right)\\ &-2\left(\sqrt{\gamma_m^{+}\bullet\gamma_s^{-}}+\sqrt{\gamma_m^{-}\bullet\gamma_s^{+}}\right)\end{aligned} \quad (5)$$

 https://doi.org/10.3390/xxxxx

$$\Delta G_{\mathrm{mws}}^{\mathrm{EL}}=\frac{\varepsilon_0\varepsilon_r\kappa}{2}\bullet\left(\zeta_m^2+\zeta_s^2\right)\left(1\text{-}\coth(h_0\kappa)+\frac{2\zeta_m\zeta_s}{\left(\zeta_m^2+\zeta_s^2\right)}\bullet\operatorname{csch}(h_0\kappa)\right) \quad (6)$$

where the subscripts m, w and s correspond to the membrane, water and SMPs/EPSs/sludge flocs, respectively. ε_0 and ε_r are the dielectric constant of the supernatant, $C^2/(m^2 \cdot N)$; h_0 is the minimum equilibrium cut-off distance, 0.158 nm. κ is the inverse Debye screening length.ζ_m and ζ_s are the surface potential of the membrane and SMPs/EPSs/sludge flocs, mV.

According to XDLVO theory, the free energy of adhesion between membrane and sludge cells/SMP can be obtained through the summation of $\Delta G_{\mathrm{mws}}^{\mathrm{LW}}$, $\Delta G_{\mathrm{mws}}^{\mathrm{AB}}$, and $\Delta G_{\mathrm{mws}}^{\mathrm{EL}}$.

$$\Delta G = G_{mws}^{LW+\Delta G_{mws}^{AB+\Delta G_{mws}^{EL}}} \quad adh \quad (7)$$

The free energy of cohesion between two surfaces of the same material, *i*, immerged in water is obtained through the summation of $\Delta G_{\mathrm{iwi}}^{\mathrm{LW}}$, $\Delta G_{\mathrm{iwi}}^{\mathrm{AB}}$, and $\Delta G_{\mathrm{iwi}}^{\mathrm{EL}}$.

$$\Delta G_{\mathrm{coh}}=\Delta G_{\mathrm{iwi}}^{\mathrm{LW}}+\Delta G_{\mathrm{iwi}}^{\mathrm{AB}}+\Delta G_{\mathrm{iwi}}^{\mathrm{EL}} \quad (8)$$

2. Interaction energy between membrane and SMPs/EPSs/sludge flocs

When the separation distance (*h*) of SMPs/EPSs/sludge flocs and membrane changes, the interaction energy per unit area between them was a function of *h.* The interaction energy per unit area between membrane and sludge flocs was based on the interaction of a flat sheet (membrane) and a sphere (SMPs/EPSs/sludge flocs), which was estimated according to the following equations:

$$U_{mws}^{XDLVO(h)=U_{mws}^{LW(h)+U_{mws}^{AB(h)+U_{mws}^{EL(h)}}}} \quad (9)$$

$$U_{mws}^{LW(h)=2\pi h_0^2\Delta G_{mws}^{LW\frac{R}{h}}} \quad (10)$$

$$U_{mws}^{AB(h)=2\pi R\lambda\Delta G_{mws}^{AB\,exp\left(\frac{h_0-h}{\lambda}\right)}} \quad (11)$$

$$U_{mws} \; EL(h)=\pi\varepsilon_0\varepsilon\left(2\zeta\, ln\left(\frac{1+e^{-\kappa h}}{1-e^{-\kappa h}}\right)_m^{2_s^2\, ln(e^{-2\kappa h}} \quad s_m\right)_r \quad (12)$$

Where *R* is the radius of the sludge flocs; λ is the characteristic decay length of AB interactions in water, which was assigned to 0.6 nm in this study.

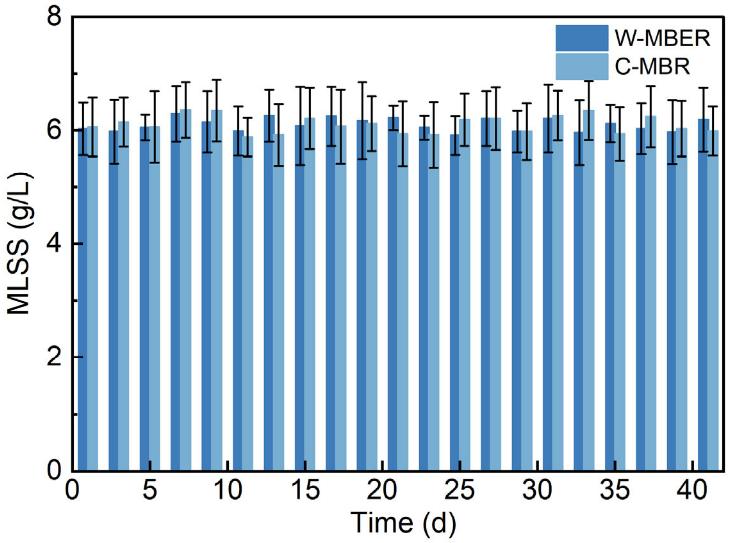

Figure S1. Variations in MLSS in the W-MBER and C-MBR.

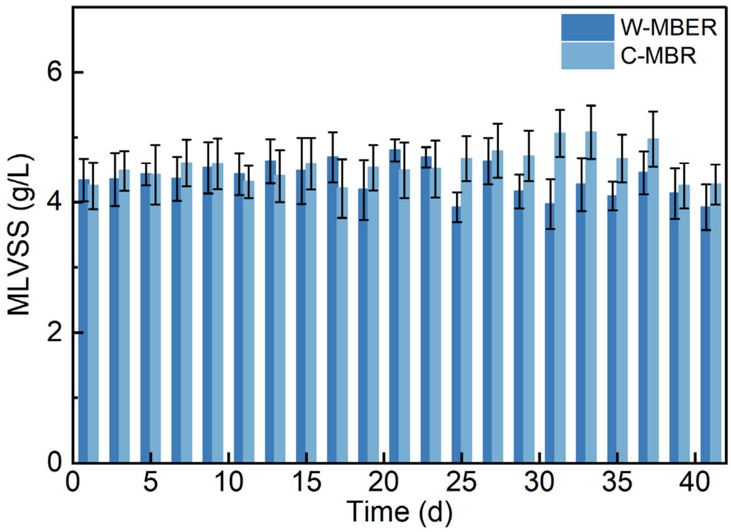

Figure S2. Variations in MLVSS in the W-MBER and C-MBR.

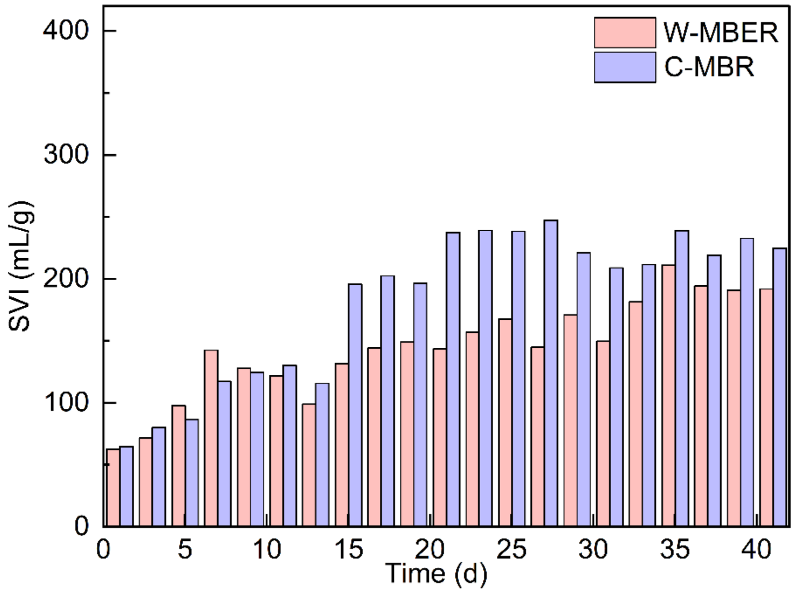

Figure S3. Variations in SVI in W-MBER and C-MBR.

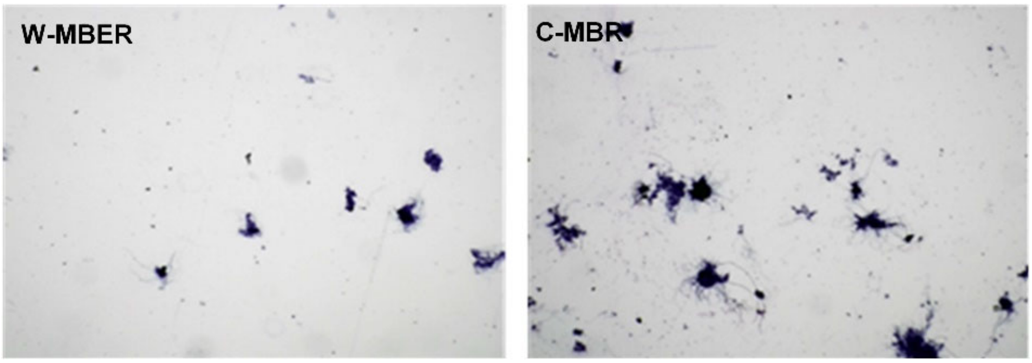

Figure S4. Sludge flocs in W-MBER and C-MBR.

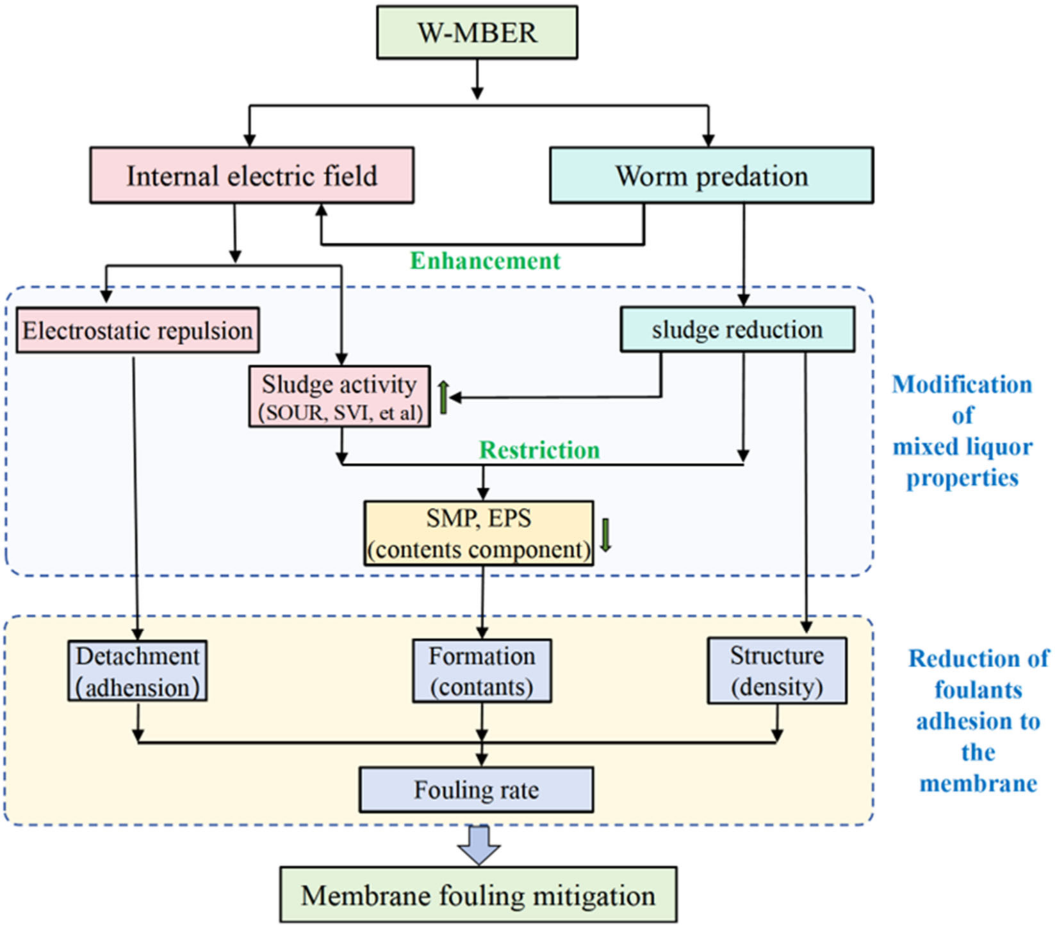

Figure S5. Schematic diagram of mechanisms of fouling mitigation in W-MBER.

Table S1. Operational timeline and conditions for worm acclimatization, reactor inoculation, and system operation phases.

Phase	Duration	Reactor Configuration	Mode	External Resistance	Key Procedures and Conditions
Worm acclimatization	15 days	Stand-alone basins	Batch-fed	Not Applicable	• Worms were acclimated in basins with activated sludge and dechlorinated tap water.<br>• DO maintained at ~1 mg/L.<br>• Sludge was replaced twice daily.<br>• Active worms were screened using a gauze method before inoculation.
Inoculation of worms and electrogenic bacteria on anode in W-MBER	3–4 weeks (Until reproducible voltage cycles achieved)	W-MBER without membrane module, aerator, and stirring magneton	Batch-fed	1000 Ω	• Screened worms were evenly distributed onto the anode.<br>• Reactor was filled with a mixture of activated sludge and synthetic wastewater.<br>• Medium was replaced when voltage dropped below 50 mV.
Long-term operation of W-MBER	42 days	W-MBER	Continuous-flow	250 Ω	• Membrane module, aerator, and stirrer were installed.<br>• Reactor was filled with aerobic activated sludge (6000 mg/L).<br>• HRT: 6 h.<br>• Membrane flux: 5.4 L/(m^2·h).<br>• Membrane cleaning performed when TMP > 30 kPa.

Table S2. Average concentration of COD, NH_4^+-N and TN in influent and effluent of W-MBER and C-MBR.

Pollutants	Influent	Effluent	
		W-MBER	C-MBR
COD (mg/L)	319.90 ± 8.50	20.58 ± 5.71	23.76 ± 7.34
NH_4^+-N (mg/L)	39.63 ± 1.36	0.55 ± 0.59	0.61 ± 1.12
TN (mg/L)	40.55 ± 0.71	25.45 ± 2.03	30.69 ± 1.66

Table S3. The membrane resistance and membrane resistance growth rate of W-MBER and C-MBR.

	W-MBER	C-MBR
R_t (10^{11}·m^{-1})	256.26	308.99
R_m (10^{11}·m^{-1})	16.60	17.42
R_f (10^{11}·m^{-1})	10.18	9.20
R_c (10^{11}·m^{-1})	229.47	282.37
R_t average growth rate (10^{11}·m^{-1}·d^{-1})	11.14	19.31
R_f average growth rate (10^{11}·m^{-1}·d^{-1})	0.44	0.58
R_c average growth rate (10^{11}·m^{-1}·d^{-1})	9.97	17.65

Table S4. Fluorescence spectral parameters of the SMP and EPS in W-MBER and C-MBR.

SMPs/EPSs	Reactor	Peak A		Peak B		Peak C		Peak D	
		Ex/Em	Intensity	Ex/Em	Intensity	Ex/Em	intensity	Ex/Em	Intensity
SMPs	W-MBER	275/326	59.13	235/334	20.07	320/388	10.79	—	—
	C-MBR	275/328	93.09	235/328	29.03	325/408	9.059	—	—
EPSs	W-MBER	280/326	416.5	230/322	239.7	365/448	38.51	270/446	50.2
	C-MBR	280/330	576.8	230/322	278	360/444	79.63	275/442	117.5

Table S5. Surface energy parameters (mJ/m^2) of clean membrane and SMPs/EPSs/sludge flocs in W-MBER and C-MBR.

Sample Description	r^{LW} (mJ/m^2)	r^{+} (mJ/m^2)	r^{-} (mJ/m^2)	r^{AB} (mJ/m^2)	r^{TOT} (mJ/m^2)	ΔG_{coh} (mJ/m^2)
Clean PVDF membrane	37.43	0.10	35.27	3.82	41.24	12.69
SMPs in W-MBER	35.42	3.11	0.65	2.85	38.27	−58.94
SMPs in C-MBR	38.60	1.52	0.92	2.36	40.96	−67.13
EPSs in W-MBER	34.62	7.59	0.11	1.83	36.46	−46.12
EPSs in C-MBR	37.16	5.98	0.10	1.56	38.72	−53.21
Sludge flocs in W-MBER	31.85	15.80	0.13	2.84	34.69	−21.95
Sludge flocs in C-MBR	32.66	7.75	0.23	2.65	35.31	−43.51

Table S6. XDLVO predictions for interaction energy (mJ/m^2) at contact between membrane and SMPs/EPSs/sludge flocs in W-MBER and C-MBR.

SMPs/EPSs	Membrane	ΔG_{mws}^{LW} (mJ/m^2)	ΔG_{mws}^{AB} (mJ/m^2)	ΔG_{mws}^{EL} (mJ/m^2)	ΔG_{adh} (mJ/m^2)
SMPs in W-MBER	PVDF membrane	−3.71	−23.54	0.11	−27.14
SMPs in C-MBR	PVDF membrane	−4.47	−28.84	0.16	−33.14
EPSs in W-MBER	PVDF membrane	−3.52	−13.31	0.11	−16.72
EPSs in C-MBR	PVDF membrane	−4.14	−16.20	0.13	−20.21
Sludge flocs in W-MBER	PVDF membrane	−2.82	−6.82	0.07	−9.57
Sludge flocs in C-MBR	PVDF membrane	−3.03	−13.29	0.07	−16.26
